# Interplay Between the IL-33/ST2 Axis and Bone Marrow ILC2s in Protease Allergen-Induced IL-5-Dependent Eosinophilia

**DOI:** 10.3389/fimmu.2020.01058

**Published:** 2020-06-02

**Authors:** Emma Boberg, Kristina Johansson, Carina Malmhäll, Jenny Calvén, Julie Weidner, Madeleine Rådinger

**Affiliations:** Krefting Research Centre, Department of Internal Medicine and Clinical Nutrition, Institute of Medicine, Sahlgrenska Academy, University of Gothenburg, Gothenburg, Sweden

**Keywords:** allergy, papain, bone marrow, eosinophilia, interleukin-33, interleukin-5, ILC2, asthma

## Abstract

**Background:** Eosinophils develop from CD34^+^ progenitor cells in the bone marrow under the influence of interleukin (IL)-5. Several cell types produce IL-5, including type 2 innate lymphoid cells (ILC2s). The alarmin cytokine IL-33 is known to activate ILC2s in mucosal tissues, but little is known about IL-33-responsive ILC2s in the bone marrow in allergen-induced airway inflammation.

**Methods:** Wild type (WT) and Rag1 deficient (*Rag1*^−/−^) mice, which lack mature T and B cells, received intranasal doses of papain to induce acute allergic inflammation. In some experiments, mice were pre-treated with anti-IL-5 prior to the papain challenge. Furthermore, recombinant IL-33 was administered to WT mice, *Rag1*^−/−^ mice, lymphocyte deficient mice (*Rag2*^−/−^*Il2rg*^−/−^) and to *ex vivo* whole bone marrow cultures. Bone marrow eosinophils and ILC2s were analyzed by flow cytometry. Eosinophil count was assessed by differential cell count and secreted IL-5 from bone marrow cells by ELISA.

**Results:** Intranasal administration of papain or IL-33 increased the number of mature eosinophils in the bone marrow despite the absence of adaptive immune cells in *Rag1*^−/−^ mice. In parallel, an increased number of eosinophils was observed in the airways together with elevated levels of Eotaxin-2/CCL24. Bone marrow ILC2s were increased after papain or IL-33 administration, whereas ILC2s was found to be increased at baseline in *Rag1*^−/−^ mice compared to WT mice. An upregulation of the IL-33 receptor (ST2) expression on bone marrow ILC2s was observed after papain challenge in both *Rag1*^−/−^ and WT mice which correlated to increased number of bone marrow eosinophilia. Furthermore, an increased number of ST2^+^ mature eosinophils in the bone marrow was observed after papain challenge, which was further dependent on IL-5. In addition, bone marrow-derived ILC2s from both mouse strains produced large amounts of IL-5 *ex vivo* after IL-33 stimulation of whole bone marrow cultures. In contrast, IL-33-induced bone marrow and airway eosinophilia were abolished in the absence of ILC2s in *Rag2*^−/−^*Il2rg*^−/−^ mice and no production of IL-5 was detected in IL-33-stimulated bone marrow cultures.

**Conclusion:** These findings establish bone marrow ILC2s and the IL-33/ST2 axis as promising targets for modulation of uncontrolled IL-5-dependent eosinophilic diseases including asthma.

## Introduction

Eosinophils are terminally differentiated granulocytes that contribute to tissue damage and remodeling in asthmatic airways by releasing toxic inflammatory mediators (1, 2). Previous studies have shown that airway allergen challenge promotes bone marrow eosinophilopoiesis in individuals with asthma and in animal models of allergic airway inflammation (3–11). Importantly, high levels of eosinophils have been shown to correlate with increasing asthma severity (12). Eosinophils develop from CD34^+^ progenitor cells in the bone marrow under the influence of the interleukin family (IL)-3, IL-5, and granulocyte-macrophage colony-stimulating factor (GM-CSF) (13, 14). IL-5 plays an essential role in eosinophil biology by controlling several key features, including terminal differentiation, proliferation, and cell migration (15–17). It was previously shown that the alarmin cytokine IL-33 induces eosinophil lineage commitment in murine bone marrow (18). This was suggested to be regulated by an IL-5-dependent mechanism since blocking IL-5 prevented IL-33-induced eosinophil expansion. However, the source of IL-5 was not addressed in the study (18). Furthermore, Anderson et al. (11) showed that murine allergen-challenge with *Alternaria* triggered IL-33 release from the airways to induce IL-5 production by lung type 2 innate lymphoid cells (ILC2s). Elevated levels of IL-5 were shown to reach the circulation and promote eosinophilopoiesis in the bone marrow (11). Indeed, ILC2s are producers of type 2 cytokines such as IL-5 and IL-13 at sites of inflammation and have been implicated in the pathogenesis of several inflammatory diseases, including asthma (19, 20). In addition, Nussbaum et al. proposed that the predominant source of circulating IL-5 is from tissue-resident ILC2s which constitutively produce IL-5 (21). Several studies have suggested that CD4^+^ T cells and CD34^+^ progenitor cells produce IL-5 locally in the bone marrow at both homeostatic conditions and after airway allergen challenge (22–24). Recently, we showed that CD34^+^ progenitors and ILC2s, but not CD4^+^ T cells produce IL-5 locally in the bone marrow of IL-33 challenged mice (25). Interestingly, bone marrow ILC2s were the predominant source of IL-5 which coincided with the expansion of IL-5-responsive CD34^+^ progenitors following IL-33 challenge (25). Indeed, a positive relationship between IL-33 and eosinophilia has been demonstrated in several studies, including reports of lower baseline levels of eosinophils in peripheral blood in knock out mice that lack IL-33 or the IL-33 receptor (ST2) (18). Moreover, studies of ST2 deficient mice in allergic inflammatory settings revealed that disruption of the IL-33 signaling pathway resulted in impaired eosinophilic airway inflammation and reduced levels of type 2 cytokines upon allergen challenge (26, 27). However, the contribution of IL-33-responsive ILC2s in allergen-induced bone marrow eosinophilia remains to be determined. Thus, in the current study we sought to assess the role of ILC2s in the regulation of allergen- and IL-33-induced bone marrow eosinophilia utilizing wild type (WT) mice, *Rag1*^−/−^ mice lacking mature T and B cells but retain ILCs, and mice lacking all lymphocytes (*Rag2*^−/−^*Il2rg*^−/−^).

## Methods

### Mice

All animal experiments were approved by the Gothenburg County Regional Ethical Committee (permit number 126/14 and 2459/19). WT C57BL/6J and C57BL/6JRj mice were purchased from Charles River (Sulzfeld, Germany) and Janvier Labs (Le Genest-Saint-Isle, France), respectively. *Rag1*^−/−^ mice and WT C57BL/6 mice, used in some experiments, were obtained from in-house breeding (University of Gothenburg, Sweden). *Rag2*^−/−^*Il2rg*^−/−^ mice were purchased from Taconic (Germantown, NJ, USA). Age and sex-matched mice at 7–12 weeks of age were used in all experiments. Mice were housed in pathogen-free conditions and were given food and water *ad libitum*.

### Allergen-Induced Airway Inflammation

To induce allergic airway inflammation mice were administered 10 μg papain (Sigma-Aldrich) intranasally on days 1–3 and samples were collected 24 hours (h) after final exposure as previously described (28). In some experiments mice received an intraperitoneal single dose of 25 μg anti-mouse IL-5 or isotype control (BD Biosciences, San Jose, CA, USA) 1 h before the first exposure. In the kinetic study, mice received an intranasal single dose of 10 μg papain and samples were collected at 3, 6, 12, 24, and 48 h. Control mice received phosphate buffered saline (PBS) vehicle.

### IL-33-Induced Airway Inflammation

IL-33-induced airway inflammation was performed as previously described (25, 29). In brief, WT, *Rag1*^−/−^, and *Rag2*^−/−^*Il2rg*^−/−^ mice were exposed to 1 μg recombinant murine IL-33 (rmIL-33, PeproTech, Rocky Hill, NJ, USA) by intranasal administration on day 1, 3, and 5. Samples were collected 24 h after the final exposure. Control mice received PBS vehicle.

### Sample Collection

Bronchoalveolar lavage (BAL), blood, and bone marrow were collected in this study. A detailed description of the sample collection is provided in Johansson et al. (29). Cells in BAL were processed for differential cell count analysis. Furthermore, cell-free BAL and serum were processed for mediator analysis by ELISA. Bone marrow cells were isolated from left and right femurs by flushing with wash buffer (2% fetal bovine serum, Sigma-Aldrich, in PBS). The samples were filtered through a 100 μm cell strainer (CellTrics®, Sysmex, Goerlitz, Germany) and red blood cells were lysed (0.1 mM EDTA in distilled water/0.15 M NH_4_Cl, Sigma-Aldrich/Merck Chemicals) by 10 minutes (min) incubation on ice. The cells were further processed for flow cytometric analysis, *ex vivo* stimulation, and differential cell count as previously described (25).

### Differential Cell Count

Approximately, 10,000–50,000 cells were used for slides (425 × g, 6 min, Shandon Cytospin 3 centrifuge) and stained with Hemacolor® Rapid stain (Merck, Darmstadt, Germany) according to the manufacturer's protocol. Eosinophils were assessed by histological examination as previously described (29).

### *Ex vivo* Stimulation of Bone Marrow Cells

Bone marrow cells from PBS exposed WT mice were seeded at a concentration of 2.5 x 10^6^/ml in complete cell culture medium: RPMI-1640 (HyClone™; GE Healthcare Life Sciences, South Logan, UT, USA), 10% fetal bovine serum (Sigma-Aldrich), 2 mM L-glutamine (HyClone), 100 U/ml penicillin, 100 μg/ml streptomycin (HyClone), 1 mM sodium pyruvate (Sigma-Aldrich). Cells were stimulated with rmIL-33 (100 ng/ml) for 24 h or kept in complete culture medium as control. Monensin (BD GolgiStop™, BD Biosciences) was added to all samples (4 μl/6 ml) during the last 3 h of the incubation. Newly produced IL-5 by ILC2s (SSC^lo^Lin^−^CD45^+^CD127^+^ST2^+^) was measured by intracellular flow cytometry. Bone marrow cells from IL-33 and PBS exposed WT mice, PBS exposed *Rag1*^−/−^ and *Rag2*^−/−^*Il2rg*^−/−^ mice were cultured as described above (2.5 × 10^6^/ml in complete cell culture medium) with or without rmIL-33 (100 ng/ml) for 44 h and cell-free culture supernatants were collected for measurement of secreted IL-5 by ELISA.

### Flow Cytometry

Bone marrow cells were resuspended in 2% mouse serum (Dako, Glostrup, Denmark) and antibodies for surface receptors were added (30 min, 4°C). Cells were washed and fixed (BD CellFix™, BD Biosciences, Erembodegem, Belgium) for 15 min in the dark at room temperature (RT). Cells were washed and analyzed on a BD FACSVerse™ Flow Cytometer running BD FACSuite™ software (BD Biosciences). Collected data were analyzed by FlowJo Software (tree Star Inc, Ashland, OR, USA). Linage negative cells were determined as CD45^+^CD3^−^CD45R/B220^−^CD11b^−^TER-119^−^Ly-G6/Gr1^−^CD11c^−^CD19^−^NK-1.1^−^FceR1^−^.

Eosinophil progenitors, mature eosinophils, and ILC2s were defined as SSC^lo^CD45^+^CD34^+^IL5Rα^+^, SSC^hi^CD45^+^CD34^−^IL5Rα^lo^CCR3+Siglec-F+, and SSC^lo^Lin^−^CD45^+^CD127^+^CD25^+^ST2^+^, respectively. Antibodies used are listed in Table S1. The IL-33 receptor (ST2) expression was estimated by mean fluorescence intensity (MFI) values. Relative MFI (rMFI) equals MFI of monoclonal antibody divided by MFI of corresponding fluorescence minus one (FMO) control.

### Intracellular Staining

Cultured bone marrow cells were stained with surface antibodies (as described above) and fixed with 4% Paraformalaldehyde (Sigma-Aldrich) in PBS for 15 min at RT in the dark. All solutions used before fixation were supplemented with Monensin (BD GolgiStop™, 4 μl/6 ml). Cells were permeabilized with 0.1% saponin (Sigma-Aldrich) in Hank's balanced salt solution (HyClone). Anti-IL-5- or isotype control antibodies were added and cells were incubated for 40 min at RT in the dark and washed before flow cytometric analysis.

### Cytokine and Chemokine Measurements

Cytokine and chemokine quantification was performed using mouse ELISA Kits (DuoSet®, R&D Systems, Minneapolis, MN, USA) according to the manufacturer's instructions. IL-5 was measured in cell-free bone marrow culture supernatants and serum. CCL24/Eotaxin-2 and IL-33 was measured in cell-free BAL. Absorbance or luminescence was measured on a Varioskan™ LUX multimode microplate reader (ThermoFisher Scientific Vantaa, Finland). Samples below detection limit was set to zero.

### Statistical Analysis

The statistical analysis was performed with GraphPad Prism 8 Software (GraphPad Software Inc, La Jolla, CA, USA). Kruskal-Wallis test was used to determine the variance among more than two groups. If significant variance was found, the nonparametric Mann-Whitney U test was used for analysis between two independent groups. Paired Student's *t*-tests were applied for the analysis of *in vitro* studies. Statistical significance was defined as ^*^P < 0.05, ^**^P < 0.01, ^***^P < 0.001 and, ^****^P < 0.0001.

## Results

### IL-33-induced Bone Marrow Eosinophilia Develops Normally in the Absence of Adaptive Immune Cells

**Figure 1:**
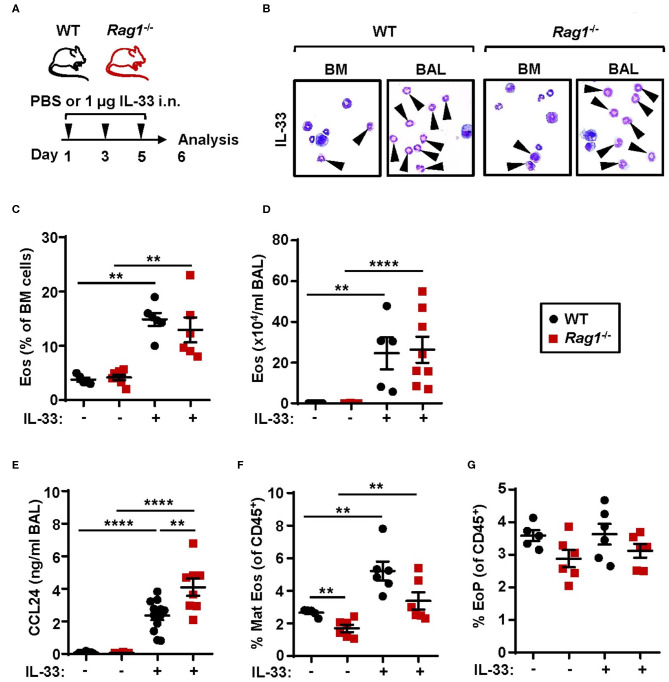
Adaptive immunity is dispensable for IL-33-induced bone marrow eosinophilia. **(A)** Wild type (WT) and *Rag1*^−/−^ mice received intranasal (i.n.) challenges of rmIL-33 or PBS vehicle and were sacrificed 24 h after the final challenge. **(B)** Representative cytospin preparations for quantification of eosinophils (eosin positive cells, indicated by arrows) in bone marrow (BM) and bronchoalveolar lavage (BAL). **(C)** Number of eosinophils (Eos) in BM and **(D)** total number of Eos in BAL analyzed by differential cell count. **(E)** Concentration of CCL24/Eotaxin-2 in BAL measured by ELISA. **(F)** Number of mature eosinophils (Mat Eos) and **(G)** eosinophil progenitors (EoPs) among all CD45^+^ BM leukocytes. Data are representative of two to four independent experiments (*n* = 5–12/group) and displayed as the mean ± SEM. Mann-Whitney U test. **P < 0.01, and ****P < 0.0001. ns, not significant.

Investigations of the requirement of ILC2s in IL-33-mediated bone marrow eosinophilopoiesis were carried out using *Rag1*^−/−^ mice which have an intact innate immune system, but lack mature T and B cells. Naïve WT and *Rag1*^−/−^ mice were challenged with rmIL-33 (Figure 1A) which resulted in increased eosinophil numbers in both bone marrow (Figures 1B,C) and BAL (Figures 1B,D) compared to control mice that received PBS vehicle (25). Furthermore, IL-33 challenge caused elevated levels of the eosinophil chemokine CCL24/Eotaxin-2 in BAL in both mouse strains where higher levels of CCL24/Eotaxin-2 were observed in *Rag1*^−/−^ mice compared to WT mice (Figure 1E). Mature eosinophils were significantly increased in the bone marrow of mice exposed to IL-33 (Figure 1F) whereas the relative number of eosinophil progenitors (Figure 1G) remained unchanged. Importantly, WT and *Rag1*^−/−^ mice responded with a similar increase of eosinophils after IL-33 challenge, suggesting that adaptive immunity is dispensable in both IL-33-driven eosinophil production and recruitment of eosinophils from the bone marrow. Thus, in the current study by using Rag*1*^−/−^ mice, we confirm our previous findings suggesting that bone marrow ILC2s support eosinophil development in WT mice in IL-33-induced inflammation (25).

### IL-33-Responsive ILC2s Produce Large Amounts of IL-5 in Both *Rag1*^-/-^ and WT Bone Marrow

**Figure 2:**
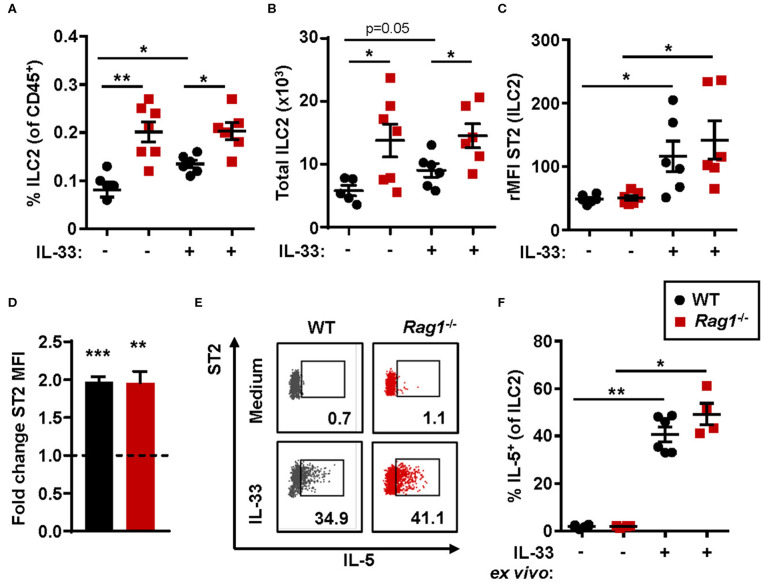
IL-33-responsive ILC2s produce IL-5 in both *Rag1*^−/−^ and WT bone marrow. **(A)** Number of type 2 innate lymphoid cells (ILC2s) among all CD45^+^ leukocytes, and **(B)** total number of ILC2s in the bone marrow. **(C)** IL-33 receptor (ST2) expression on ILC2s showed as relative mean fluorescence intensity (rMFI) in the bone marrow of wild type (WT) and *Rag1*^−/−^ mice exposed to IL-33 or PBS control. **(D)** Representative dot plots of IL-5^+^ ILC2s following *ex vivo* stimulation with IL-33 or unstimulated medium controls (values indicate percent of the parent population). **(E)** Fold change ST2 MFI (MFI of IL-33 stimulated cells divided by MFI of unstimulated control cells). **(F)** Number of IL-5^+^ cells among ILC2s. Data are representative of two to four independent experiments (*n* = 4–7/group) and displayed as the mean ± SEM. Mann-Whitney U test and paired *t*-test **(D)**. *P < 0.05, **P < 0.01, and ***P < 0.001. ST2 = IL-33 receptor.

The number of bone marrow ILC2s in *Rag1*^−/−^ mice were significantly increased when compared to WT controls (Figures 2A,B) at baseline. Thus, IL-33 challenge resulted in a significant increase of ILC2s in WT bone marrow only, and not in bone marrow from *Rag1*^−/−^ mice (Figure 2A). Moreover, ILC2s from both mouse strains responded to IL-33 challenge with increased ST2 expression (Figure 2C). Of note, 100% of bone marrow ILC2s (SSC^lo^Lin^−^CD45^+^CD127^+^CD25^+^) in PBS and IL-33 challenged mice were ST2^+^, while IL-33 challenge further increased the ST2 receptor expression (Figure 2C). Furthermore, these results were consistent with an approximate 2-fold upregulation of ST2 expression evaluated after IL-33 stimulation of WT and *Rag1*^−/−^ bone marrow cells *ex vivo* (Figure 2D). In addition, stimulation with IL-33 in *ex vivo* cultures generated high levels of IL-5^+^ ILC2s in both mouse strains (Figures 2E,F), which suggests that bone marrow ILC2s contribute to IL-33-induced eosinophil development *in vivo* independent of adaptive immunity.

### Intranasal Administration of Papain Induces Bone Marrow Eosinophilia in *Rag1*^–/–^ Mice

**Figure 3:**
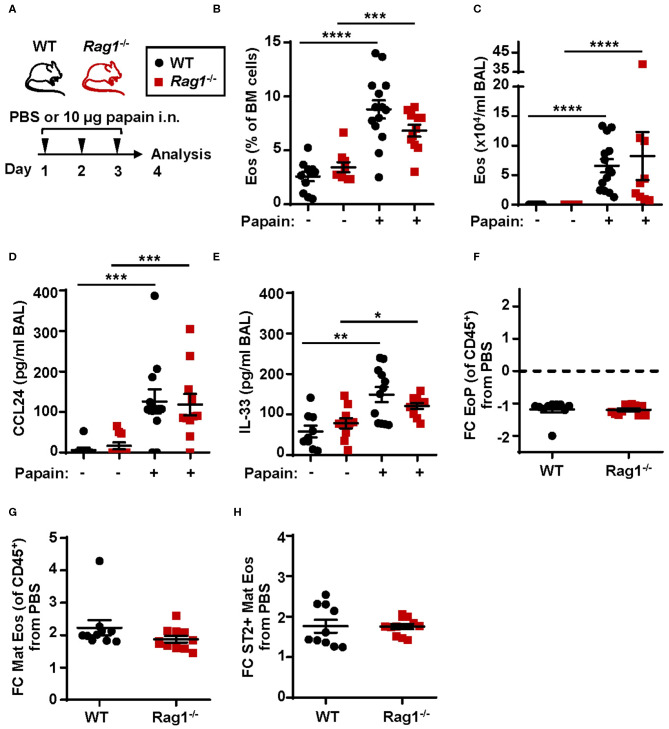
Papain induced IL-33-driven eosinophilic inflammation is similar in *Rag1*^−/−^ and WT mice. **(A)** Wild type (WT) and *Rag1*^−/−^ mice received intranasal (i.n.) challenges of papain or PBS vehicle and were sacrificed 24 h after the final challenge. **(B)** Number of eosinophils (Eos) in bone marrow (BM) and **(C)** total number of Eos in BAL analyzed by differential cell count. **(D)** Concentration of CCL24/Eotaxin-2 and **(E)** IL-33 in BAL measured by ELISA. **(F)** Fold change (FC) in numbers of eosinophil progenitors (EoPs), **(G)** mature eosinophils (Mat Eos) and **(H)** ST2^+^ Mat Eos among all CD45^+^ BM leukocytes in mice exposed to papain compared to PBS control. Data are representative of at least three independent experiments (*n* = 7–14/group) and displayed as the mean ± SEM. Mann-Whitney U test. *P < 0.05, **P < 0.01, ***P < 0.001, and ****P < 0.0001. ST2 = IL-33 receptor.

To investigate eosinophil development and the downstream effects of IL-33 in allergic inflammation WT and *Rag1*^−/−^ mice were subjected to a model of allergic inflammation using the protease allergen, papain (Figure 3A). Increased numbers of eosinophils in both bone marrow (Figure 3B) and BAL (Figure 3C) were detected in WT and *Rag1*^−/−^ mice following papain challenge. Furthermore, both WT and *Rag1*^−/−^ mice demonstrated elevated levels of CCL24/Eotaxin-2 and IL-33 in BAL following papain challenge (Figures 3D,E) allowing mature eosinophils to migrate toward the airways. A marginally decreased relative number of eosinophil progenitors was observed (Figure 3F and Figure S1A) whereas an approximate 2-fold increase of mature eosinophils was found in papain-challenged WT and *Rag1*^−/−^ mice compared to saline exposed control mice (Figure 3G and Figure S1B). We have previously reported a higher amount of ST2^+^ mature bone marrow eosinophils in IL-33 induced inflammation (25). We next investigated the amount of ST2^+^ mature bone marrow eosinophils during allergic inflammation and whether adaptive immunity is required for the generation of ST2-expressing eosinophils. An increase of ST2^+^ mature eosinophils was detected in WT and importantly, elevated levels of ST2^+^ mature eosinophils were also detected in papain challenged *Rag1*^−/−^ mice (Figure 3H and Figure S1C). Altogether, these results indicate that adaptive immunity is not required for induction of papain-driven eosinophilic inflammation in the bone marrow where IL-33 is an important mediator.

### Protease Allergen Challenge Induces an Increased ST2 Expression on Bone Marrow ILC2s Which Correlates to Bone Marrow Eosinophilia

**Figure 4:**
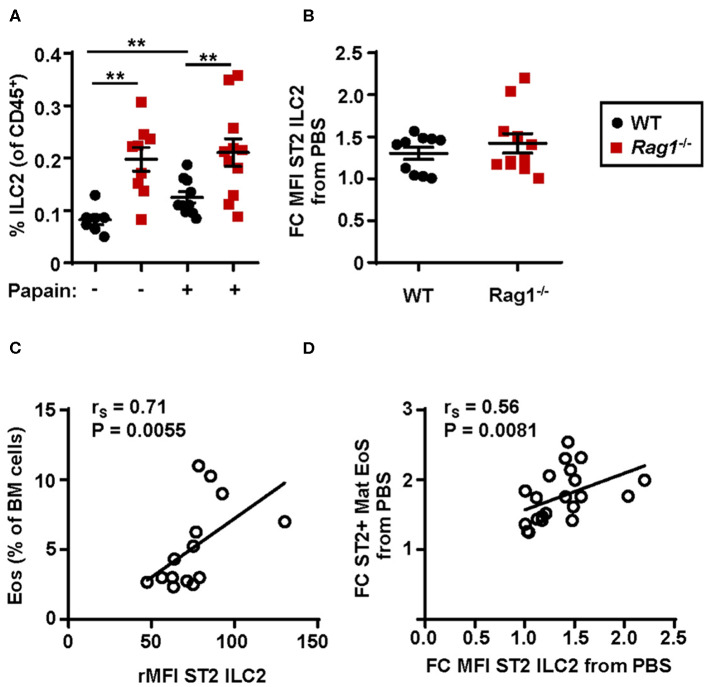
Protease allergen challenge makes bone marrow ILC2s susceptible to IL-33 by upregulation of the ST2 receptor. **(A)** Number of type 2 innate lymphoid cells (ILC2s) among all CD45^+^ leukocytes, and **(B)** fold change MFI (mean fluorescence intensity) IL-33 receptor (ST2) expression on ILC2s in the bone marrow (BM) of wild type (WT) and *Rag1*^−/−^ mice exposed to papain compared to PBS control. **(C)** Correlation plot of relative MFI (rMFI) ST2 on ILC2s and the number of BM eosinophils (Eos) in WT and *Rag1*^−/−^ mice from two representative experiments **(D)** Correlation plot of ST2^+^ mature eosinophils and ST2 expression on ILC2s in the BM of papain challenged WT and *Rag1*^−/−^ mice compared to PBS controls presented as fold change (FC). Data are representative of three independent experiments unless stated otherwise (*n* = 7–11/group) and shown as the mean ± SEM. Correlation were performed by using Spearman's rho, with r_S_ indicating the Spearman correlation coefficient. Mann-Whitney U test. **P < 0.01. ST2 = IL-33 receptor.

An increased relative number of bone marrow ILC2s was observed in WT mice airway challenged with papain compared to PBS exposed control mice whereas *Rag1*^−/−^ mice had a higher number of bone marrow ILC2s already at baseline (i.e., PBS exposed control) (Figure 4A). Bone marrow ILC2s from both WT and *Rag1*^−/−^ mice upregulated ST2 expression after papain challenge, which is crucial for the activation of ILC2 effector functions (Figure 4B). Moreover, high ST2 expression on ILC2s demonstrated a positive correlation with both increased bone marrow eosinophilia (Figure 4C) and an increased number of ST2^+^ mature eosinophils (Figure 4D). Collectively, these data indicate that IL-33-responsive bone marrow ILC2s contribute to allergen-induced bone marrow and airway eosinophilia.

### Bone Marrow ILC2s Upregulate ST2 Expression Within 24 h After Airway Papain Challenge

**Figure 5:**
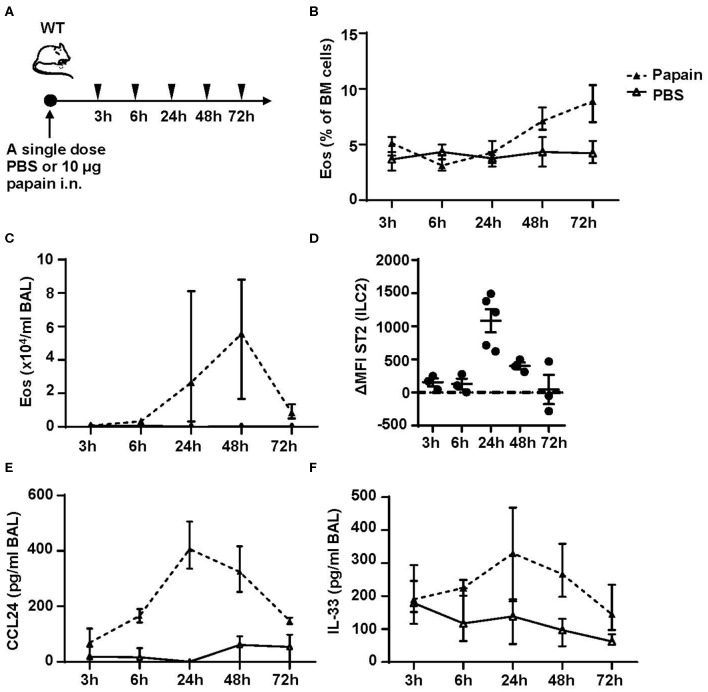
Kinetic profile of bone marrow ILC2s and eosinophilia post protease allergen challenge. **(A)** Wild type (WT) mice received one dose PBS or papain intranasally (i.n.) and were sacrificed at 3, 6, 24, 48, and 72 h. **(B)** Number of eosinophils (Eos) in bone marrow (BM) and **(C)** total number of Eos in BAL analyzed by differential cell count. **(D)** IL-33 receptor (ST2) expression on ILC2s shown as delta mean fluorescence intensity (ΔMFI) in the BM of wild type (WT) mice challenged with papain compared to PBS vehicle mice where PBS equals zero (dotted line). **(E)** Concentration of CCL24/Eotaxin-2 and **(F)** IL-33 in BAL measured by ELISA. Data are representative of one experiment (*n* = 3–5/group) and displayed as the mean ± SEM **(D)** or mean with min/max values represented by error bars **(B,C,E,F)**.

We next performed a time course to identify when bone marrow ILC2s respond to the inhaled protease allergen papain. WT mice were exposed to a single dose of papain intranasally (Figure 5A). Elevated levels of eosinophils in bone marrow were detected at 48 h (Figure 5B) whereas a higher relative number of BAL eosinophils was seen at 24 h (Figure 5C). In line with increased eosinophil levels in the airways there was a significant increase of ST2 expression on ILC2s in the bone marrow (Figure 5D). Additionally, high levels of BAL CCL24/Eotaxin-2 (Figure 5E) and a trend toward elevated IL-33 levels in BAL (Figure 5F) were detected at this time point. At 48 h, the ST2 expression on bone marrow ILC2s was significantly lower and CCL24/Eotaxin-2 and IL-33 levels in BAL showed a trend toward decreased levels. At 72 h the inflammation in the airways was resolved where BAL eosinophil levels were similar to control mice (Figure 5C). In line with these data, the ST2 expression on bone marrow ILC2s was significantly decreased along with lower BAL CCL24/Eotaxin-2 and IL-33 levels. These data suggest that eosinophils are no longer recruited to the airways at 72 h post airway challenge. Collectively, our results suggest that bone marrow ILC2s act rapidly in response to an inhaled allergen by upregulating its ST2 receptor expression in the bone marrow at the onset of eosinophil development.

### Mature Eosinophils are Unable to Develop in Absence of ILC2s in *Rag2*^–/–^*Il2rg*^–/–^ Bone Marrow

**Figure 6:**
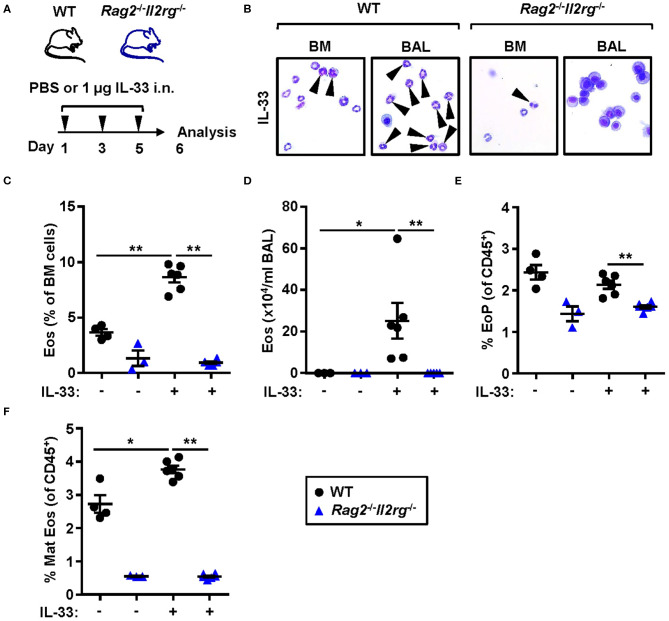
Lymphocyte deficient mice are unable to develop IL-33-induced bone marrow and BAL eosinophilia. **(A)** Wild type (WT) and *Rag2*^−/−^*Il2rg*^−/−^ mice received intranasal (i.n.) challenges of rmIL-33 or PBS vehicle and were sacrificed 24 h after the final challenge. **(B)** Representative cytospin preparations for quantification of eosinophils (eosin positive cells, indicated by arrows) in bone marrow (BM) and bronchoalveolar lavage (BAL). **(C)** Number of eosinophils (Eos) in BM and **(D)** total number of Eos in BAL analyzed by differential cell count. **(E)** Number of eosinophil progenitors (EoPs) and **(F)** mature eosinophils (Mat Eos) among all CD45^+^ BM leukocytes. Data are representative of one experiment (*n* = 3–6/group) and displayed as the mean ± SEM. Mann-Whitney U test. *P < 0.05 and **P < 0.01.

To determine whether IL-33-induced eosinophilia can develop in the absence of ILC2s, we challenged naïve lymphocyte deficient *Rag2*^−/−^*Il2rg*^−/−^ mice with rmIL-33 (Figure 6A). No induction of eosinophils was detected by differential cell count analysis of the bone marrow (Figures 6B,C) and BAL (Figures 6B,D) samples from *Rag2*^−/−^*Il2rg*^−/−^ mice. The relative number of eosinophil progenitors was lower in *Rag2*^−/−^*Il2rg*^−/−^ bone marrow compared to WT (Figure 6E). The most dramatic difference was observed in the number of mature eosinophils which was severely impaired in the bone marrow of both IL-33 and PBS challenged *Rag2*^−/−^*Il2rg*^−/−^ mice (Figure 6F). Together, these data suggest that innate lymphocytes are essential for the maintenance of eosinophils under homeostatic conditions as well as for the maturation of eosinophils in response to inflammatory signals.

### Absence of the Adaptive Immune System Results in Higher Levels of IL-5 in IL-33-Induced Inflammation

**Figure 7:**
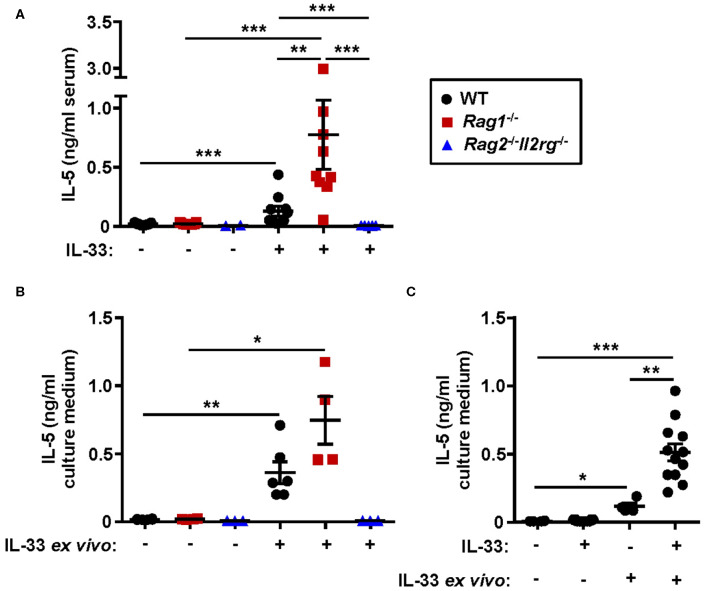
Absence of IL-5 in IL-33 challenged lymphocyte deficient mice. **(A)** Concentration of IL-5 in serum of wild type (WT), *Rag1*^−/−^ and *Rag2*^−/−^*Il2rg*^−/−^ mice exposed to IL-33 or PBS. **(B)** IL-5 in culture medium of bone marrow (BM) cells stimulated with rmIL-33 (100 ng/ml) for 44 h or unstimulated medium controls. **(C)** IL-5 in the culture medium of BM cells from IL-33 challenged or PBS controls (*in vivo*) followed by restimulation with rmIL-33 (100 ng/ml) *ex vivo* for 44 h or unstimulated medium controls. Data are representative of four independent experiments (*n* = 2–12/group) and shown as the mean ± SEM. Mann-Whitney U test and paired *t*-test **(B)**. *P < 0.05, **P < 0.01, and ***P < 0.001.

Both systemic IL-5 and local IL-5 production have been suggested to regulate eosinophil development and maturation in the bone marrow (11, 21, 25). Next, we analyzed the concentration of IL-5 in serum from WT, *Rag1*^−/−^ and *Rag2*^−/−^*Il2rg*^−/−^ mice. IL-33 challenge significantly increased levels of IL-5 in serum of WT and *Rag1*^−/−^ mice but not in serum of *Rag2*^−/−^*Il2rg*^−/−^ mice (Figure 7A). Notably, the level of serum IL-5 was higher in IL-33 challenged *Rag1*^−/−^ mice compared to WT mice (Figure 7A), which might be associated with a greater number of ILC2s in the bone marrow of *Rag1*^−/−^ mice. Furthermore, bone marrow cultures from WT and *Rag1*^−/−^ mice stimulated with IL-33 accumulated IL-5 in the culture media, but no IL-5 was detected in cultures from *Rag2*^−/−^*Il2rg*^−/−^ mice (Figure 7B). Interestingly, we found that bone marrow cultures generated from *in vivo* IL-33 challenged mice produced significantly more IL-5 upon IL-33 re-stimulation *ex vivo* (Figure 7C). This result suggests that in IL-33 challenged mice presenting an increased expression of ST2 on bone marrow ILC2s makes the cells more susceptible to IL-33, thus, potentially leading to a higher production of IL-5, which in turn drives eosinophil development.

### IL-5 is Required for Allergen-Induced Eosinophilic Inflammation Despite the Absence of an Adaptive Immune System

**Figure 8:**
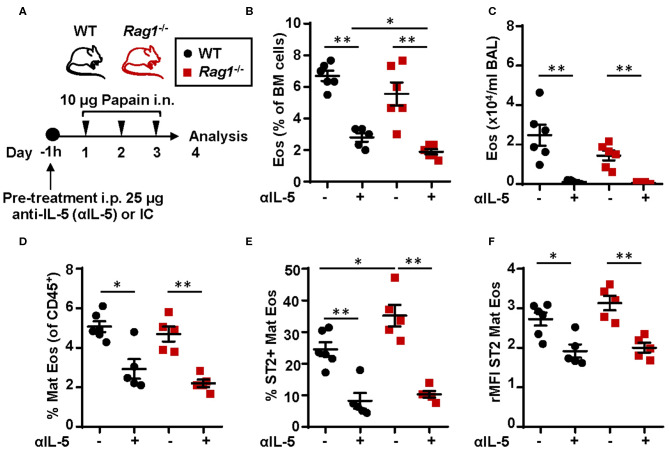
Neutralization of IL-5 disrupts ST2 expression on bone marrow eosinophils and abolish protease allergen-induced eosinophilic inflammation **(A)** Wild type (WT) and *Rag1*^−/−^ mice were pre-treated with anti-IL-5 (αIL-5) or isotype control (IC), received intranasal (i.n.) challenges of papain and sacrificed 24 h after the final challenge. **(B)** Number of eosinophils (Eos) in bone marrow (BM) and **(C)** total number of Eos in BAL analyzed by differential cell count. **(D)** Number of mature eosinophils (Mat Eos) among all CD45^+^ BM leukocytes. **(E)** Number of ST2^+^ Mat Eos in BM. **(F)** ST2 expression on BM Mat Eos shown as relative mean fluorescence intensity (rMFI). Data are representative of three independent experiments (*n* = 4–6/group) and displayed as the mean ± SEM. Mann-Whitney U test. *P < 0.05, **P < 0.01. IC is isotype control. ST2 = IL-33 receptor.

To assess whether IL-5 is required in the development and maturation of bone marrow eosinophilia in *Rag1*^−/−^ and WT mice, mice were pre-treated with anti-IL-5 antibodies 1 h before the dose regimen of intranasal papain exposures (Figure 8A). Anti-IL-5 treated papain challenged *Rag1*^−/−^ and WT mice were unable to induce eosinophilia in the bone marrow (Figures 8B,D) and BAL (Figure 8C). Moreover, a decreased number of ST2^+^ mature eosinophils was seen in both mouse strains treated with anti-IL-5 (Figure 8E). Interestingly, a significant decrease of the ST2 expression on mature eosinophils was observed in both *Rag1*^−/−^ and WT mice (Figure 8F). These findings suggest link between IL-5 levels and ST2 expression on eosinophils in the bone marrow. The relative number of eosinophil progenitors (data not shown) and ILC2s were not affected by anti-IL-5 treatment (Figure S2). Collectively, these data show that papain induced eosinophilic inflammation is dependent on IL-5 and that IL-5 levels might regulate ST2 expression on bone marrow eosinophils which in turn drives the maturation of eosinophil within the bone marrow. In addition, the absence of IL-5-producing T cells in *Rag1*^−/−^ mice make bone marrow ILC2s a potential local source of IL-5.

## Discussion

A growing body of evidence links IL-33 to initiating events that occur during allergen exposure. Important findings include the identification of IL-33 and ST2 as major susceptibility loci in several genome-wide association studies of allergic diseases (30–35). In addition, a loss-of-function mutation in the *Il33* gene has been reported to be associated with lower number of blood eosinophils and a reduced risk of developing asthma (36). Moreover, vaccination against IL-33 reduced airway eosinophilia and airway hyperresponsiveness in a mouse model of HDM-induced asthma, propose IL-33 as a promising target for asthma intervention (37). A major finding in our study is that ST2^+^ ILC2s in the bone marrow is activated upon one intranasal dose of the protease allergen papain within 24 h. This was followed by an increased number of eosinophils in the bone marrow and airways. Many studies of the ILC2 potent alarmin IL-33 in allergic diseases have focused on barrier tissues such as the lung, but our findings suggest that IL-33 has additional roles in the bone marrow. Analysis of ILC2s in naïve mice show that bone marrow-derived ILC2s exhibit a 6-fold higher ST2 expression compared to lung-derived ILC2s (29). It is possible that the expression level of ST2 is related to differential stages of ILC2 development in the bone marrow (i.e., ILC2 precursors) (38) vs. the lung, but more importantly, it indicates that IL-33 signaling plays a role in both compartments. Constantly exposed to the inhaled environment, ILC2s in the airway mucosa are indeed located in a prime position to react to IL-33. The bone marrow on the other hand is privileged in this respect and the high ST2 expression may suggest that bone marrow ILC2s are more sensitive to IL-33, thus even low levels of IL-33 might be enough to activate the cells at a distance.

Several immune cells express ST2 including eosinophils and can thereby produce proinflammatory mediators upon activation (39). We show that both the number of mature eosinophils in the bone marrow and the ST2 expression on these cells increases upon protease allergen challenge (Figures 3G,H). These results are in line with a recent study demonstrating an increased number of ST2-expressing sputum and blood eosinophils post 24 h allergen challenge in allergic asthmatics (40). Furthermore, our study show that the ST2 expression was dependent on IL-5 demonstrated by the use of anti-IL-5 treatment (Figures 8E,F) suggesting a link between IL-5 and ST2 expression on eosinophils. Prospective studies have to be carried out in order to determine whether IL-5 directly or indirectly regulate the ST2 expression on eosinophils. We further demonstrate that ST2^+^ mature eosinophils positively correlate with an increased ST2 receptor expression on ILC2s, suggesting both cell types being IL-33-responsive simultaneously in allergen-induced eosinophilic inflammation. Previous studies have investigated IL-33-responsive blood eosinophils in atopic subjects in terms of effects on adhesion, degranulation and chemotaxis (39, 41). Eosinophils were more adherent and potent in degranulation upon IL-33 stimulation whereas no effects was observed on chemotaxis (39, 41). Moreover, the IL-33/ST2/IL-5 axis was recently shown to influence eosinophilopoiesis where ST2 deficient mice challenged with the fungal Alternaria exhibited lower levels of IL-5 in serum and a decreased relative number of eosinophils in the bone marrow (11). In addition, it has been shown that the increased number of IL-5^+^ blood eosinophils from allergic asthmatics observed after IL-33 stimulation was attenuated by treatment with either anti-ST2 monoclonal antibody, soluble ST2 or the combination of the two (40). An emerging interest in targeting IL-33 and its receptor ST2 with pharmacological treatments in individuals with respiratory diseases including asthma can be seen with examples such as SAR440340 (ClinicalTrials.gov identifier, NCT03546907) from Regeneron Pharmaceuticals and Sanofi, MSTT1041A (ClinicalTrials.gov Identifier: NCT02918019) from Hoffmann-La Roche, GSK3772847 (ClinicalTrials.gov Identifier: NCT03207243) from GlaxoSmithKline and etokimab (ClinicalTrials.gov Identifier: NCT03469934) from AnaptysBio. Moreover, the anti-IL-33 or anti-ST2 strategy may hold a potential clinical future outside the field of respiratory diseases with allergy or atopic dermatitis as examples.

Interestingly, we observed an increased relative number of bone marrow ILC2s and an upregulation of ST2 expression on these cells after intranasal challenges with the allergen protease papain. These findings were also observed in IL-33 challenged mice which are in line with previous reports (42–44). In contrast, Stier et al. recently reported a decreased number of progenitor ILC2s in the bone marrow upon acute exposure to the fungal allergen *Alternaria* (45). This process was indeed dependent on IL-33 signaling as both ST2 and IL-33 deficient mice demonstrated lower numbers of ILC2s in peripheral tissues while progenitor ILC2s accumulated in the bone marrow (45). Furthermore, we have unpublished data revealing an increased ST2 expression on bone marrow ILC2s in HDM-sensitized mice, thus strengthen a role for bone marrow ILC2s at an early stage of eosinophilic inflammation caused by a variety of allergens. In line with our results, Brickshawana et al. showed that IL-33 stimulation increased ST2 expression and IL-5 production in an early study of bone marrow ILC2s (42). However, the functional implication of IL-33-responsive ILC2s in the development of eosinophilia was not addressed in their study.

Importantly, we identified IL-5^+^ bone marrow ILC2s in IL-33 induced inflammation and we demonstrate by neutralizing IL-5 *in vivo*, that IL-5 is essential in eosinophilic inflammation in the bone marrow in response to the protease allergen papain. We further demonstrate that bone marrow ILC2s are able to respond within 24 h by increased ST2 expression to an intranasal single dose of papain. At 48 h, the ST2 expression on ILC2s was starting to decrease and simultaneously eosinophils were increased in the bone marrow. In addition, a similar increased relative number of mature bone marrow eosinophils was found after papain challenge in *Rag1*^−/−^ mice lacking adaptive immune cells, but with an intact innate immune system including functional ILC2s. Therefore, bone marrow ILC2s, in addition to airway ILC2s, are a possible early source of IL-5 in allergic diseases including asthma (46). Likewise, restimulation with IL-33 *ex vivo* revealed that bone marrow cultures from IL-33 exposed mice produced larger amounts of IL-5 compared to bone marrow cultures from PBS control mice (Figure 7C). Indeed, it has previously been discovered that allergen-experienced airway ILC2s responded more vigorously to a second challenge compared to naïve ILC2s (47) and that ILC2s previously exposed to papain or rmIL-33 displayed higher responsiveness even to unrelated allergens (47). Innate immune memory is an emerging field with important implications in ILC2 biology that might extend to ILC2 functions in the bone marrow, with potential consequences such as increased eosinophil hematopoiesis in allergic subjects (4, 7, 9, 48).

In our continued analysis of the requirement of ILC2s in IL-33-driven eosinophilic inflammation, we demonstrate for the first time that *Rag1*^−/−^ mice display normal eosinophil development in the bone marrow in both direct IL-33- and in protease allergen-induced eosinophilic inflammation. This result eliminates a contribution from the adaptive immune system during acute airway inflammation and adds a functional effect of ILC2s in the bone marrow. Interestingly, we observed a significant increased relative number of ILC2s in the bone marrow of *Rag1*^−/−^ mice compared to WT mice. In addition, IL-33 challenged *Rag1*^−/−^ mice also demonstrated higher levels of serum IL-5 and CCL24/Eotaxin-2 in BAL compared to WT. These excessive responses might reflect a lack of inhibitory signals that are mediated by adaptive immune cells and should be further assessed in prospective studies. For instance, ST2^+^ T regulatory cells have been described to be superior to ST2^−^ T regulatory cells in suppressing type 2 inflammation (49). Further, *Rag1*^−/−^ mice also exhibited a wide range of bone marrow ILC2s at both baseline and upon airway challenge. These data might indicate heterogeneity in the ILC2 population.

A striking result in our study was that *Rag2*^−/−^*Il2rg*^−/−^ mice displayed a reduced number of bone marrow eosinophils at baseline and were unable to develop bone marrow eosinophilia in response to IL-33 challenge. These findings clearly show that innate lymphocytes are essential for the development of eosinophils and we found it interesting that the difference was more apparent in the number of mature eosinophils compared to eosinophil progenitors, which might reflect the critical role of IL-5 for terminal differentiation and maturation of eosinophils. Indeed, no IL-5 production was detected in bone marrow cultures from *Rag2*^−/−^*Il2rg*^−/−^ mice and the level of serum IL-5 was below detection limit. Moreover, we show that *Rag2*^−/−^*Il2rg*^−/−^ mice are unable to develop airway eosinophilia, which is consistent with a previous study where airway eosinophil numbers were restored upon adoptive transfer of ILC2s to *Rag2*^−/−^*Il2rg*^−/−^ mice (28).

We conclude that IL-33 plays important roles for the initiation of bone marrow eosinophilia where IL-33-responsive bone marrow ILC2s contribute to allergen-induced IL-5-dependent eosinophilic inflammation. Identification of mechanisms that regulate eosinophilic inflammation is critical for developing new therapies of uncontrolled eosinophilic asthma.

## Data Availability Statement

The datasets generated for this study are available on request to the corresponding author.

## Ethics Statement

The animal study was reviewed and approved by Gothenburg County Regional Ethical Committee Kammarrätten i Göteborg Box 1531 401 50 Gothenburg Sweden.

## Author Contributions

EB, KJ, and MR designed experiments and wrote the manuscript. EB, KJ, JW, JC, MR, and CM performed the experiments and approved the final manuscript. EB and KJ analyzed data. JW, JC, and CM provided comments to the manuscript.

## Conflict of Interest

The authors declare that the research was conducted in the absence of any commercial or financial relationships that could be construed as a potential conflict of interest.

## Supplementary Material

Figure S1 **(A)** number of eosinophil progenitors (EoPs) and **(B)** mature eosinophils (Mat Eos) among all CD45^+^ BM leukocytes. **(C)** Number of ST2^+^ Mat Eos in BM. Data are representative of three independent experiments (*n* = 7–11/group) and displayed as the mean ± SEM. Mann-Whitney U test. ^*^*P* < 0.05, ^***^*P* < 0.001, and ^****^*P* < 0.0001. ST2 = IL-33 receptor.

Figure S2 Number of type 2 innate lymphoid cells (ILC2s) among all CD45^+^ leukocytes in wild type (WT) and *Rag1*^−/−^ mice pre-treated with anti-IL-5 (αIL-5) or isotype control (IC) 1 h prior to intranasal (i.n.) challenges of papain (Figure 8A).

Table S1 Antibodies used in flow cytometry.

## Abbreviations

BAL: Bronchoalveolar lavage
ILC2: type 2 innate lymphoid cell
IL: Interleukin
WT: Wild type.
