# Supplementary figures and images for: Interplay Between the IL-33/ST2 Axis and Bone Marrow ILC2s in Protease Allergen-Induced IL-5-Dependent Eosinophilia
Source: Front Immunol. 2020 Jun 2;11:1058. doi: 10.3389/fimmu.2020.01058 (PMC7280539; doi:10.3389/fimmu.2020.01058)

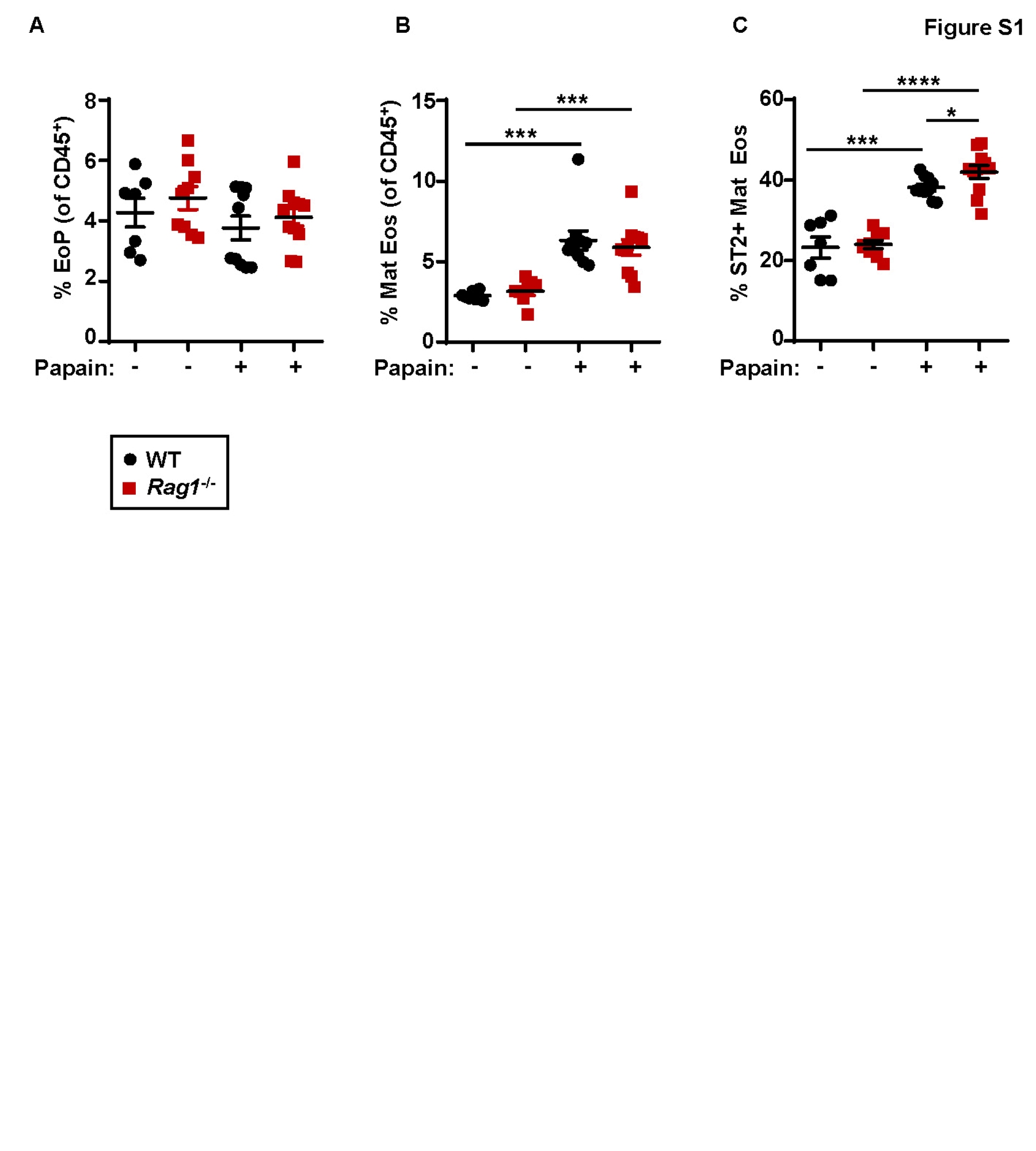

Supplement: Figure S1 — (A) number of eosinophil progenitors (EoPs) and (B) mature eosinophils (Mat Eos) among all CD45+ BM leukocytes. (C) Number of ST2+ Mat Eos in BM. Data are representative of three independent experiments (n = 7–11/group) and displayed as the mean ± SEM. Mann-Whitney U test. *P < 0.05, ***P < 0.001, and ****P < 0.0001. ST2 = IL-33 receptor. [file Image_1.jpeg]

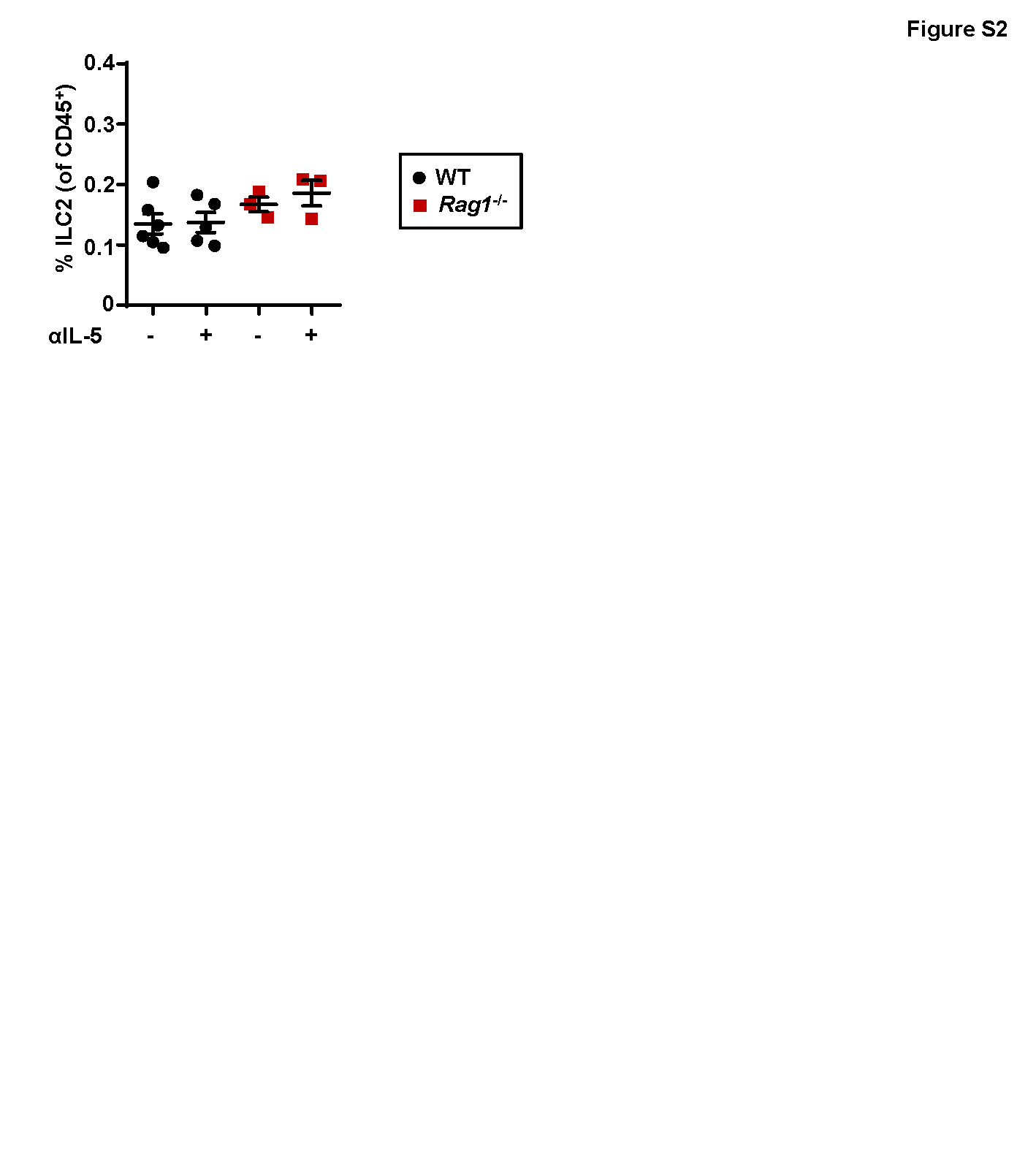

Supplement: Figure S2 — Number of type 2 innate lymphoid cells (ILC2s) among all CD45+ leukocytes in wild type (WT) and Rag1−/− mice pre-treated with anti-IL-5 (αIL-5) or isotype control (IC) 1 h prior to intranasal (i.n.) challenges of papain (Figure 8A). [file Image_2.jpeg]
