# Supplementary material for: Interplay Between the IL-33/ST2 Axis and Bone Marrow ILC2s in Protease Allergen-Induced IL-5-Dependent Eosinophilia
Source: Front Immunol. 2020 Jun 2;11:1058. doi: 10.3389/fimmu.2020.01058 (PMC7280539; doi:10.3389/fimmu.2020.01058)
Supplement: Table S1 — Antibodies used in flow cytometry. [file Table_1.pdf]

Supplemental Table 1

Antigen (clone)	Format	Manufacturer
**Hematopoietic Lineage cocktail: CD3 (17A2), CD45R/B220 (RA3-6B2), CD11b (M1/70), TER-119 (TER-119), Ly-G6/Gr-1 (RB6-8C5)**	FITC	ThermoFisher Scientific
**CD11c (N418)**	FITC	ThermoFisher Scientific
**CD19 (1D3)**	FITC	BD Bioscience
**NK-1.1 (PK136)**	FITC	BD Bioscience
**FceR1 (MAR-1)**	FITC	ThermoFisher Scientific
**CD34 (RAM34)**	FITC	BD Bioscience
**CD127/IL-7Ra (A7R34)**	PE	ThermoFisher Scientific
**CD125 (T21)**	PE	BD Bioscience
**CD45 (30-F11)**	PerCP	BD Bioscience
**CD25 (PC61)**	APC	BD Bioscience
**ST2/IL-33R (RMST2-2)**	APC	ThermoFisher Scientific
**Siglec-F (E50-2440)**	AF647	BD Bioscience
**CD25/IL-2RA (PC61)**	BV421	BD Bioscience
**CD193/CCR3 (J073E5)**	BV421	BioLegend
**IL-5 (TRFK5)**	BV421	BioLegend
**Isotype control IL-5/Rat IgG1, κ (RTK2071)**	BV421	BioLegend
